# A Systematic Review of Chemotherapeutic Regimens Used in Pancreatic Cancer

**DOI:** 10.7759/cureus.46630

**Published:** 2023-10-07

**Authors:** Nimra Awais, Travis Satnarine, Areeg Ahmed, Ayesha Haq, Deepkumar Patel, Grethel N Hernandez, Kofi D Seffah, Mustafa Abrar Zaman, Safeera Khan

**Affiliations:** 1 Research, California Institute of Behavioral Neurosciences and Psychology, Fairfield, USA; 2 Pediatrics, California Institute of Behavioral Neurosciences and Psychology, Fairfield, USA; 3 Internal Medicine, California Institute of Behavioral Neurosciences and Psychology, Fairfield, USA; 4 Family Medicine, California Institute of Behavioral Neurosciences and Psychology, Fairfield, USA; 5 Internal Medicine, Piedmont Athens Regional, Athens, USA; 6 Internal Medicine, St. George's University School of Medicine, Newcastle Upon Tyne, GBR

**Keywords:** first and second line chemotherapy, folfirinox, gemcitabine, nab-paclitaxel, palliative chemotherapy, pancreatic cancer

## Abstract

Pancreatic cancer is a malignant tumor with one of the worst prognosis. Its incidence has been on the rise in recent years. First-line and second-line treatments as well as adjuvant therapies have been employed in clinical trials for pancreatic cancer along with traditional chemotherapy and radiotherapy that has been enhanced. The prognosis of pancreatic ductal adenocarcinoma (PDAC) is still quite bad despite recent improvements in diagnostic and treatment methods. Since most patients are not candidates for treatment with a curative purpose, effective palliative care is crucial. For this systematic review, between December 25, 2022, and January 5, 2023, we searched PubMed, Medline, Cochrane, and Science Direct and discovered 225 relevant articles. The appropriateness of the literature abstracts for the pooled analysis was evaluated using different combinations of keywords such as pancreatic cancer, first- and second-line chemotherapy, palliative chemotherapy, gemcitabine and nab-paclitaxel (GnP), FOLFIRINOX (FFX), and fluorouracil. Eight research studies with a total of 15,236 people, including systematic reviews, meta-analyses, and randomized controlled trials (RCTs), were included. The only treatment of choice for patients without metastatic disease who have clinical staging that suggests resectable or borderline resectable pancreatic cancer (BRPC) should be resection. This research examined how first- and second-line chemotherapeutic regimens (using different drug combinations) affected patients with locally advanced pancreatic cancer (LAPC) or BRPC and how they responded in terms of overall survival (OS), tumor resectability, and progression-free interval. The review concludes by highlighting the results of these therapies. Notably, a growing body of research indicates that the two most popular first-line medication combinations GnP and FFX have similar results in RCTs and in real-world populations. Results of second-line therapy after first-line regime failure are still dismal, and there is still a great deal of doubt regarding the best course of action. More RCTs and real-world evidence studies that address current and innovative regimens, as well as the best order in which to administer them, are required, with a greater emphasis on targeted therapy with fewer side effects.

## Introduction and background

In 2017, there were 447,665 new cases of pancreatic cancer reported worldwide (58.6 per million), with a prevalence of 49.8 per million and 441,083 fatalities (57.7 per million). Only patients who can undergo a complete resection have a chance of cure [1]. For patients who have metastatic disease, median survival is often less than half that when the disease is unresectable due to invasion into important arterial structures. Although 5% to 10% of individuals have either a significant family history of pancreatic cancer, a recognizable mutation that increases risk, or both, most pancreatic tumors lack a genetic predisposition [1]. Chronic pancreatitis, obesity, type 2 diabetes, excessive consumption of red meat, alcohol abuse, and smoking are risk factors [2].​Palliative systemic therapy and/or radiotherapy are the sole available treatments for patients as well as for those resected patients who experience relapses [1].

Neoadjuvant chemotherapy (NAC) should be considered for individuals with borderline resectable and locally advanced pancreatic ductal adenocarcinoma (PDAC). This neoadjuvant treatment idea is supported by recent randomized controlled trials (RCTs). Neoadjuvant FOLFIRINOX (FFX) therapy and radio-chemotherapy with gemcitabine both yielded encouraging outcomes [2]. NAC has been utilized to raise the R0 resection (surgical margin is microscopically negative for residual tumor) rate and transform locally progressed, unresectable cancers into tumors that may be resectable as a result of the recent development of more effective chemotherapy regimens [2]. Additionally, NAC benefits patients by treating early micrometastatic illness, enhancing survival rates, and choosing poor responders who advance on treatment prior to surgery, sparing them from a pointless procedure. According to these factors, various studies have shown that NAC is superior to initial surgery for patients with borderline resectable pancreatic cancer (BRPC) and locally advanced pancreatic cancer (LAPC). As a result, the National Comprehensive Cancer Network also suggests NAC as a conventional treatment for BRPC and LAPC [2].

In the past, 5-fluorouracil (5-FU)-based regimens were the only options for palliative therapy of PDAC, and they typically produced at best minimal outcomes. However, evidence from a randomized study conducted in 1996 revealed that palliative chemotherapy in PDAC increased median overall survival (MOs) as well as quality of life when compared to the best supportive care alone [3]. This study randomized 90 patients to chemotherapy or best supportive care. Results showed that chemotherapy improved quality of life more often than best supportive care (10%, 4/41). More patients in the chemotherapy group had a higher quality of life for at least four months (36%, 17/49). Overall survival (OS) was longer in the chemotherapy group, and quality-adjusted survival time was longer for patients randomized to chemotherapy [3]. At the time, 5-FU treatment was more or less experimental. Based on the findings of a subsequent randomized trial with prolonged MOs in favor of gemcitabine, gemcitabine took the position of 5-FU as the industry standard in this clinical scenario the following year [4]. In this study, we'll contrast how this regimen affects patients with BRPC and LAPC. When compared to single-agent gemcitabine, multi-agent regimens like FFX and gemcitabine and nab-paclitaxel (GnP) have demonstrated considerable benefits. When compared to gemcitabine alone, both regimens had survival times that were twice as long and response rates of about 30% [4]. This study found that 23.8% of gemcitabine-treated patients experienced clinical benefit, with median survival durations of 5.65 and 4.41 months, respectively. The 12-month survival rate was 18% for gemcitabine patients and 2% for 5-FU patients [4].

The success of the combination therapy will be aided by improving our knowledge of the molecular basis of pancreatic cancer and discovering more potent, targeted systemic treatments [5]. Recent advancements in sequencing technology have led to the development of molecular targeted therapy and immunotherapy for pancreatic cancer. These therapies target mutated genes and pathways contributing to tumorigenesis. Immunotherapy has shown promising results in various cancer types but has faced challenges due to the complex and immunosuppressed microenvironment of pancreatic cancer. However, immunotherapy showed encouraging results when paired with other treatments to cause immunoreactions [5]. Enhancing preventive strategies and early identification can help the surgical outcomes as we work to overcome the formidable challenge of pancreatic cancer. Likewise, combination therapy, such as standard therapy (surgery, radiotherapy, or chemotherapy) combined with target therapy and immunotherapy, should also show favorable outcomes in the treatment of pancreatic cancer [4,5].

The purpose of this research was to compare the current treatments (first-line and second-line gemcitabine/gemcitabine-based regimes) for BRPC or LAPC. We focused on OS as primary outcome, progression-free survival (PFS), and resection rate of tumor (R0) as secondary outcomes after receiving these therapies. Additional research is needed with regard to the side effects of these chemotherapeutic medications and targeted therapy with fewer side effects that can produce more encouraging gains.

## Review

Methods

This systematic review was conducted using the Preferred Reporting Items for Systematic Reviews and Meta-Analysis (PRISMA) 2020 guidelines [6].

Search Sources and Strategy

We searched PubMed, Cochrane Library, Medline, and ScienceDirect for relevant literature. Various combinations of keywords like pancreatic cancer, first- and second-line chemotherapy, palliative chemotherapy, gemcitabine and nab-paclitaxel (GnP), FOLFIRINOX (FFX), and fluorouracil were used to search all databases. In PubMed, along with these keywords, the following strategy was used for the relevant search: ("pancreatic neoplasms/drug therapy"[Majr] OR "pancreatic neoplasm fluorouracil"[Mesh] AND "gemcitabine"[Mesh] neoplasms/therapy"[Majr]) AND "FOLFIRINOX."

Table 1 summarizes the databases used and the number of articles identified from that database.

**Table 1:** Databases used and identified number of papers *An additional 12 records were identified through supplementary searches, such as scanning reference lists of included articles, bringing the total after filters to 225. MESH: Medical Subject Headings

Search strategy	Database used	Number of papers identified	Number of papers after applying filters
Pancreatic cancer AND gemcitabine AND chemotherapy	PubMed	1,893	124
("Pancreatic Neoplasms/drug therapy"[Majr] OR "Pancreatic Neoplasm Fluorouracil"[Mesh] AND "Gemcitabine"[Mesh] Neoplasms/therapy"[Majr]) AND "FOLFIRINOX"	MESH	199	76
Pancreatic cancer AND gemcitabine AND fluorouracil	Cochrane library	2	0
Pancreatic cancer AND gemcitabine AND fluorouracil	Medline	10	0
Pancreatic cancer AND chemotherapy AND palliative chemotherapy	ScienceDirect	166	13
Total		2,270	225*

Eligibility criteria

*Inclusion* *Criteria and Exclusion Criteria*

We incorporated the latest publications and articles published in the past five years (the study focus was to include recent advancements the reason a five-year filter was used), including papers written in the English language. We only included research papers involving human participants with diagnosed BRPC or LAPC. If the entire contents of a research paper could not be accessed, it was excluded. Non-accessible papers were excluded due to a lack of institutional subscription and financial resources. A list of our inclusion and exclusion criteria is shown in Table 2.

**Table 2:** Inclusion and exclusion criteria​ BRPC: borderline resectable pancreatic cancer, LAPC: locally advanced pancreatic cancer

Inclusion criteria	Exclusion criteria
Papers written and published in the English language in the past five years	Grey literature
Type of intervention included any form of chemotherapy, any combination of chemotherapy and radiotherapy, best supportive care, or another chemotherapy and/or radiotherapy treatment plan. Papers focusing on patients with pancreatic cancer who received gemcitabine and fluorouracil (as first- and second-line treatment) with BRPC or LAPC	Papers written and published in languages other than English
Research papers involving human participants	If the complete text of the papers could not be retrieved, articles were excluded

Characteristics of participants

People with a diagnosis of pancreatic adenocarcinoma were established by either histological or cytological findings (investigations on body tissue or cells). Studies enrolling people with advanced, unresectable, or recurrent disease were eligible for inclusion.

Data extraction

A Microsoft Excel spreadsheet (Microsoft Corporation, Washington, United States) was used to extract the data. The study design, sample size, objectives, use of chemotherapy or radiation therapy as an intervention, and conclusions were used to categorize the eligible studies. Both primary (OS) and secondary outcomes (R0, PFS, and OS) were assessed. After a comprehensive article review and extract, primary and secondary outcomes were evaluated. NA independently extracted data using a specified form.

Selection process

After eliminating the duplicate papers, we moved the articles to the Endnote. Titles and abstracts were used to screen each article by NA (the first author independently). Only pertinent articles were analyzed when the entire texts of the shortlisted articles were assessed. Only articles that met the inclusion and exclusion criteria were given a chance to be shortlisted.

Quality assessment of studies

We used the assessment of multiple systematic reviews (AMSTAR) checklist for systematic reviews and meta-analysis and the Cochrane risk of bias tool used for RCTs to assess the risk of bias.

Data collection process

Following the finalization of the articles for the systematic review and extraction, the primary outcomes and other pertinent data were evaluated. The data was separately collected by NA, and the following details were gleaned: authorship, publication year, study design, study objective population, intervention employed, result, and conclusion.

Results

**Figure 1:**
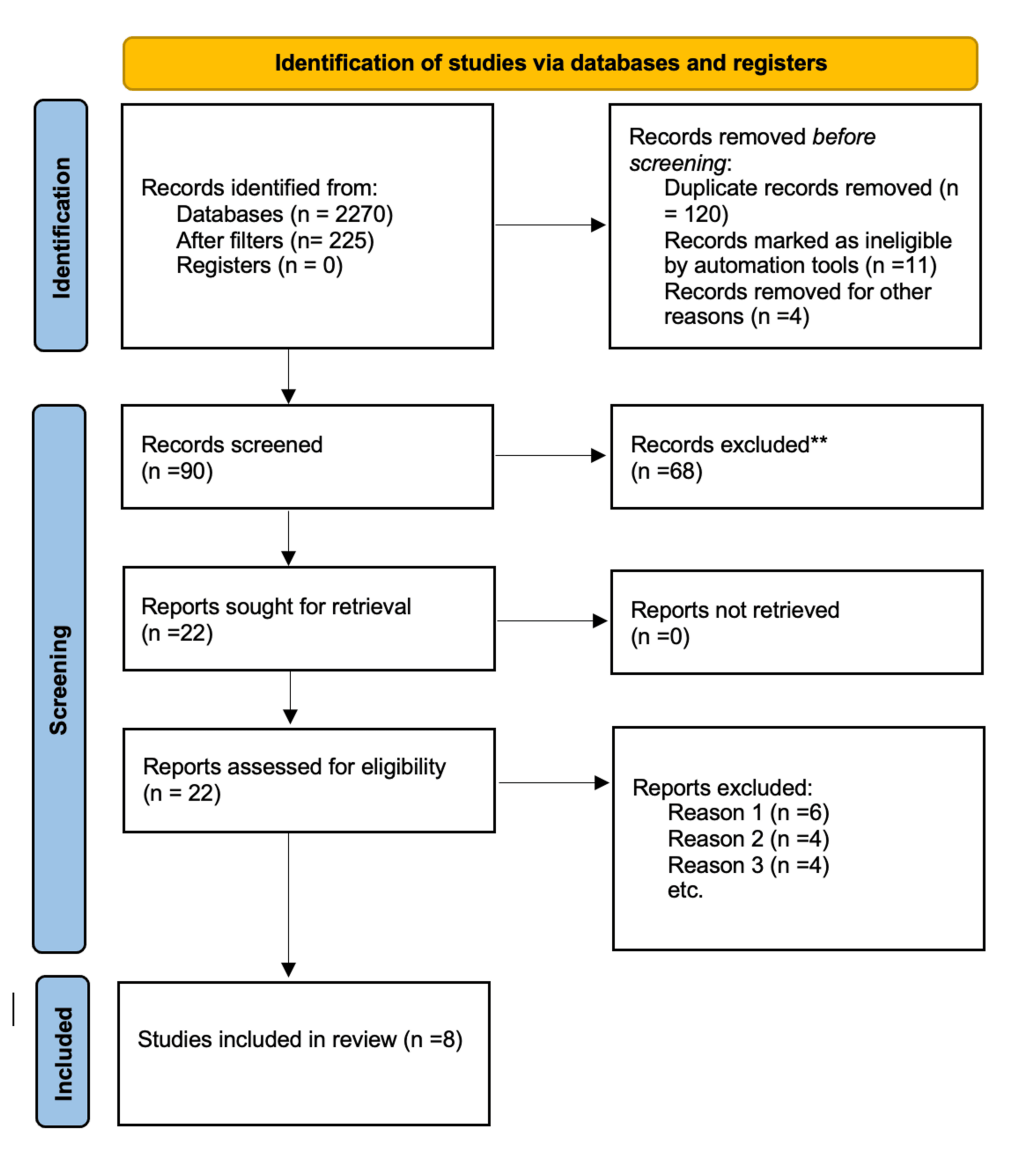
PRISMA flowchart illustrating the article selection process Reason 1: paper was irrelevant. Reason 2: unable to access the paper. Reason 3: did not satisfy inclusion criteria. MESH: Medical Subject Headings, PRISMA: Preferred Reporting Items for Systematic Reviews and Meta-Analysis

A total of 2,270 publications were gathered via PubMed, Medline, ScienceDirect, and Cochrane Library. There were 90 articles left after the duplicates were removed, and they were filtered according to the inclusion and exclusion criteria. After the screening and eligibility, only eight pertinent journal articles remained. An overview of the PRISMA flow chart is shown in Figure 1.

Study Identification and Selection

Eight published journal articles that included 15,236 patients underwent thorough selection analysis. There were four RCTs and four systematic reviews and meta-analyses. The pertinent articles included in this review are summarized in Table 3. The patient's health, illness stage, drug adverse effects (AE), prior chemotherapeutic treatment, and resistance to it are all important considerations when choosing the regimen.

**Table 3:** Summary of the studies included IRI: irinotecan, FP: fluoropyrimidine, OXA: oxaliplatin, 5-FU: fluorouracil, S1: oral 5-FU, OS: overall survival, PFS: progression-free survival, QoL: quality of life, FFX, FOLFIRINOX, GnP: gemcitabine and nab-paclitaxel, mPAC: metastatic pancreatic adenocarcinoma, BRPC: borderline resectable pancreatic cancer, LAPC: locally advanced pancreatic carcinoma, FA: folinic acid, mFFX: modified FOLFIRINOX, DFS: disease-free survival, RCT: randomized controlled trial, MOs: median overall survival, AE: adverse effects

Author and year of publication	Type of study	Number of participants	Drugs used	Result	Conclusion
Dong et al., 2022 ​[2]	Systematic review and meta-analysis	1,351	FFX, GnP	FFX had improved PFS and OS, a greater resection rate (including R0 resection rate), and a lower rate of severe toxicity. Between the two groups, there are no differences in the rates of stable disease or partial/complete regression	In comparison to the GnP group, the FFX group reported higher resection and R0 resection rates as well as superior PFS and OS results for patients with BRPC and LAPC. Compared to GnP, there was no higher rate of serious toxicity for FFX
Citterio et al., 2018 [7]	Meta-analysis	1,587	IRI, FP, FA, and OXA. IRI, fluoropyridine, cisplatin, and OXA	In terms of FP and OXA-FP, in particular, the combination IRI-FP-FA outperformed all other therapies in terms of OS and PFS	Patients who had not previously received treatment with IRI-FP-FA may benefit from OS when used as a second-line treatment for metastatic pancreatic cancer
Go et al., 2021​ [8]	RCT	80	mFFX, OXA, IRI, leucovorin, and 5-FU infusion	In the mFFX and S-1 groups, the median PFS rates were 5.2 and 2.2 months In the mFFX and S-1 groups, the MOs rates were 9.2 and 4.9 months. In the mFFX and S-1 groups, 56% and 17% of the patients experienced grade 3-4 adverse events	For mPAC patients resistant to gemcitabine-based chemotherapy, the administration of mFFX as a second-line chemotherapy treatment led to higher survival rates than S-1 treatment alone
Villaorduña et al., 2022 ​[9]	Clinical trial	22	FFX and gemcitabine-based chemoradiation	Eighteen of the 22 patients received. In patients who had at least one cycle of FFX, the bias-corrected R0 rate was 55.6%, and in patients who underwent surgery, it was 80%. The OS had a median of 35.1 months. Thirty-four months was the median PFS for patients who underwent surgery	High R0 resection rates were achieved in patients undergoing surgery when a multimodality treatment combining FFX, gemcitabine, and radiation was used
Fietkau et al., 2020​ [10]	Randomized trial	180	Gemcitabine and FFX chemoradiotherapy	About 126/180 individuals who underwent induction chemotherapy were randomly assigned to receive further treatment. Thirty-six of the 126 individuals who received trial therapy underwent surgery. Patients with R0 resected tumors had significantly higher DFS and OS rates than non-operated patients	After receiving neoadjuvant therapy, pancreatic cancer tumor resectability that was staged as unresectable at the original diagnosis should be reevaluated. If resectability is established, surgery should be performed on the patient because it vastly improves their prognosis
Assenat et al​., 2021 [11]	Clinical trial	91	Gembrax (GnP)​ and FFX	In 3.5% of patients, the tumor completely responded, in 61.4% it only partially responded, and in 19.3% of patients, it progressed. The median PFS and OS were 15.1 months and 10.5 months, respectively	Sequential GnP and FFX demonstrated acceptable toxicity as first-line therapy with no limiting neurotoxicity while demonstrating high response and survival rates
Pusceddu et al., 2019 [12]	Systematic review and meta-analysis	3,813	GnP and FFX	The mean weighted OS difference was in favor of FFX by 1.15 months. Between the two arms, PFS did not differ. When compared to GnP and FFX, the total response rate was comparable (25 vs. 24%). As compared to FFX, GnP had less neurotoxicity and anemia, while FFX had less nausea, neutropenia, and febrile neutropenia	No significant advantage of one regimen over the other in terms of overall risk of death and progression, despite FFX having a longer median OS compared to GnP
Chin et al., 2018 [13]	Systematic review	9,463	5-FU, FFX, gemcitabine, platinum, topoisomerase inhibitor, nab-paclitaxel, gemcitabine incorporating (GEMOXEL or cisplatin/epirubicin/5-FU/gemcitabine)	5-FU reported lower OS, PFS, and QoL compared to gemcitabine alone. In terms of OS, PFS, and response rates, FFX outperformed gemcitabine, but it was associated with more adverse events. When compared to bolus dosage, gemcitabine administration at a fixed dose rate improved OS but increased the rate of adverse events. As compared to gemcitabine alone, combinations with platinum enhanced PFS and response rates but not OS. The frequency of adverse reactions grew. Fluoropyrimidine added to gemcitabine enhanced OS, PFS, and response rates but significantly raised AE. Topoisomerase inhibitor plus gemcitabine did not boost survival rates but did increase toxicity. GnP enhanced side effects while improving OS, PFS, and response rates. OS, PFS, and QoL were all improved by multidrug regimens incorporating gemcitabine (GEMOXEL or cisplatin/epirubicin/5-FU/gemcitabine)	The long-established standard of care, gemcitabine, has recently been replaced by combination chemotherapy. It is quite effective to combine FFX with gemcitabine and GnP. Clinicopathological categorization is still tricky, which makes choosing the best treatment for certain patients challenging. To assist patients in making informed treatment decisions, biomarker discovery is crucial

The shortlisted articles were evaluated for quality assessment using the relevant techniques. The Cochrane risk of bias tool was used to evaluate RCTs, while the AMSTAR checklist was used to evaluate systematic reviews and meta-analyses. The quality assessment used to evaluate study bias is shown in Table 4-5.

**Table 4:** AMSTAR checklist for a systematic review and meta-analysis AMSTAR: assessment of multiple systematic reviews Q1- Did the research questions and inclusion criteria for review include the components of PICO? Q2 - Did the report of the review contain an explicit statement that review methods were established prior to the conduct of the review and did the report justify any significant deviations from the protocol? Q3 - Did the review authors explain their selection of the study designs for inclusion in the review? Q4 - Did the review authors use a comprehensive literature search strategy? Q5 – Did the review authors perform study selection in duplicate? Q6 - Did the review authors perform data extraction in duplicate? Q7 - Did the review authors provide a list of excluded studies and justify the exclusions? Q8 - Did the review authors describe the included studies in adequate detail? Q9 - Did the review authors use a satisfactory technique for assessing the risk of bias (Rob) in individual studies that were included in the review? Q10 - Did the review authors report on the sources of funding for the studies included in the review? Q11 - If meta-analysis was performed, did the review authors use appropriate methods for statistical combinations of results? Q12 - If meta-analysis was performed, did the review authors assess the potential impact of RoB in individual studies on the results of the meta-analysis or other evidence synthesis? Q13 - Did the review authors account for RoB in individual studies when interpreting/discussing the results of the review? Q14 - Did the review authors provide a satisfactory explanation for and discussion of any heterogeneity observed in the results of the review? Q15 - If they performed quantitative synthesis, did the review authors carry out an adequate investigation of publication bias and discuss its likely impact on the results of the review? Q16 - Did the review authors report any potential sources of conflict of interest, including any funding they received for conducting the review?

AMSTAR checklist	Dong ​ et al​., 2022 [2]	Citteria et al., 2018 ​[7]	Pusceddu et al., 2019 [12]	Chin et al., 2018 [13]
Q1	Yes	Yes	Yes	Yes
Q2	Yes	No	Yes	Yes
Q3	Yes	Yes	Yes	Yes
Q4	Yes	Yes	Yes	Yes
Q5	Yes	Yes	Yes	Yes
Q6	No	No	No	No
Q7	No	Yes	No	No
Q8	Yes	Yes	Yes	Yes
Q9	No	Yes	Yes	Yes
Q10	Yes	No	Yes	Yes
Q11	Yes	Yes	Yes	Yes
Q12	No	Yes	Yes	Yes
Q13	Yes	Yes	Yes	Yes
Q14	Yes	Yes	Yes	No
Q15	Yes	No	Yes	No
Q16	Yes	Yes	Yes	No

**Table 5:** Risk of bias assessment for RCTs RCTs: randomized control trials

Study type​ (RCTs)	Selection bias​ (randomization process)	Allocation concealment	Performance bias	Detection bias	Attrition bias	Reporting bias	Other bias
Go et al., 2021 ​[8]	Yes	Yes	No	No	Yes	Yes	Unclear
Villaorduna et al., 2022l​ [9]	Yes	Unclear	No	Unclear	Yes	Yes	No
Fietkau et al., 2020 ​[10]	Yes	Yes	No	Yes	Yes	Yes	No
Assenat et al​., 2021 [11]	Yes	Yes	Yes	Yes	Yes	Yes	Unclear

Discussion

One of the most challenging cases for medical oncologists worldwide continues to be pancreatic cancer that cannot be surgically removed. Pancreatic cancer is resistant to radiation and chemotherapy because of its hypoxic microenvironment. The variability of the genes or the expression of the targets also contributes to its resistance to conventional therapy. These are some challenges faced while treating pancreatic cancer [5]. Although diagnostic imaging tools have improved, the majority of cases are still discovered at a stage when there is no chance of recovery or long-term survival. However, there are valid grounds for cautious hope [5].

In this study, we review the outcomes of clinical trials and observational studies that discuss various chemotherapy regimens used in patients with LAPC or BRPC and how those patients respond in terms of OS, PFS, and resection rate following chemotherapy.

First- and Second-Line Therapy

The first-line therapy has been the subject of numerous studies, while research on the second-line is still scarce. For the best-performing patients, the FFX regimen combining 5-FU, oxaliplatin (OXA), irinotecan (IRI), and folinic acid (FA) is used as an alternative to the standard first-line regimens of combinations of gemcitabine-based chemotherapy, such as gemcitabine + nab-paclitaxel (FA). Making a decision between these two regimens presents a difficult dilemma because, in order for patients to receive second-line chemotherapy, the option is premised on their prior treatment [7].

IRI-containing treatment plans (IRI-FP-FA, IRI-FP, and IRI) were the most successful therapy combinations when it came to treating OS. IRI-FP-FA was the best treatment in terms of PFS, followed by OXA-FP-FA plus IRI. The OXA-FP and FP combination had the lowest outcomes in this scenario [7]. The median distribution was around 85% for OS and close to 55% for PFS. The combination IRI-FP-FA showed superior performance compared to other treatments, particularly for FP and OXA-FP, in terms of OS and PFS. The most effective therapeutic combinations were IRI-FP-FA, followed by IRI-FP (58%) and IRI. The best treatment for PFS was IRI-FP-FA (90%), followed by OXA-FP-FA plus IRI [7]. Based on OS and PFS assessment, Citterio et al. suggested FP, FA, and IRI formulation for the second-line treatment following the first-line gemcitabine/gemcitabine combination.

Due to its invasive biological properties, chemotherapy is the cornerstone of advanced pancreatic cancer. The selection of the first-line therapy for metastatic pancreatic cancer is influenced by a wide range of variables. Relapse-free survival duration is important in deciding between the two treatments, in case of relapse following adjuvant gemcitabine or gemcitabine-based therapy, with GnP only being useful in cases of patient relapse occurring after six months. Since triplet therapy has a higher toxicity profile than GnP, the decision to use FFX is primarily based on the health of the patient. Additionally, hospitalization for supportive care, the use of pegfilgrastim, and the cost of anti-emetics associated with FFX toxicity should all be taken into account when choosing a course of treatment. Compared to FFX, GnP therapy is less expensive, with costs primarily associated with drug acquisition [8,9]. Apart from patient age, health, stage of the disease, and the costs of anticancer drugs as well as general management expenses like hospital stays or prescription expenditures for AE control must be taken into account when analyzing the financial aspects of anticancer treatment. In order to receive the FFX regimen, patients must be admitted to a hospital. The cost of extended hospital stays, the administration of granulocyte colony-stimulating factor, and infection control (associated with febrile neutropenia) are also more likely to occur in patients receiving FFX due to the increased hematologic AE rates [8,9].

Recent encouraging results with FFX in neoadjuvant and adjuvant settings suggest that this treatment may eventually move from the first line to these settings. In light of the rising number of patients with LAPC or resected pancreatic cancer who will receive FFX, GnP may soon be viewed as the best first-line therapy [9].

For patients with BRPC and LAPC, multi-agent chemotherapy regimens like FFX and GnP have demonstrated considerable benefits when compared to single-agent gemcitabine. It is still debatable if FFX and GnP are effective and safe NACs for BRPC and LAPC [10].

Villaorduna et al. reported that patients with borderline resectable PDAC responded effectively to the multimodality regimen using induction FFX, gemcitabine, and radiation. The R0 resection rate was 55.6%, and the regimen was well tolerated. In addition, 80% of patients had R0 margins and 68.2% were surgical candidates. With a one-year OS rate of 85.1% across all trial participants, the median OS reached close to three years. FFX outperformed GnP in terms of resectability rater and R0 resection rate, PFS and OS, and severe toxicity rate while having greater resection rates overall. There were no differences between the two groups in the rates of stable disease or partial/complete regression. For patients with BRPC and LAPC, the FFX group outperformed the GnP group in terms of resection and R0 resection rates, as well as PFS and OS outcomes. When compared to GnP, FFX did not exhibit a higher rate of serious toxicity [9]. The GABRINOX (GnP followed by FFX) response rate, safety, and efficacy were evaluated in a phase Ib-II trial in 2021. GnP was administered sequentially, and it was hypothesized that targeting the tumor microenvironment with nab-paclitaxel would improve FFX access to the tumor and, consequently, its efficacy. In addition to a higher survival rate, this phase Ib-II research demonstrated high response rates, acceptable tolerability, and no neurotoxicity [12].

Role of Multimodality Therapy

In patients with borderline resectable PDAC, a sequential multimodality therapy consisting of induction FFX, concurrent gemcitabine, and radiation showed good effectiveness with an R0 resection rate of 55.6% and was well tolerated. The induction phase of a cancer treatment involved FFX, a regimen of OXA, leucovorin, IRI, and 5-FU. The regimen was given every two weeks for four cycles, with the response assessed by CT. Patients without progression of disease (POD) proceeded to the chemoradiation phase, while those with POD continued management. Chemoradiation was given within six weeks, using external beam radiation therapy and intensity-modulated radiation therapy to a total dose of 50.4 Gy (the gray (Gy) is the unit of ionizing radiation) in 28 fractions. Patients received weekly gemcitabine dosed at 400 mg/m2. After four to six weeks, the treatment response was evaluated by imaging and a multidisciplinary board. Patients with resectable disease underwent surgery [9]. As 68.2% of the enrolled patients were able to undergo surgery, this regimen demonstrated a favorable balance between efficacy and tolerance; the other patients were unable to tolerate the treatment or developed metastatic illness. Another indicator of effectiveness is the high rate (80%) of patients having surgery with R0 margins, with 100% of them being recurrence-free after one year. Despite the R0 resection rate, this regimen is effective because the median OS is close to three years and the one-year OS rate is 85.1% across all trial participants [9].

Phase two of an incremental multimodality regime investigation was conducted based on 22 individuals with tumors that had metastases in four or more regional lymph nodes and were limited to the pancreas, head, and neck. Induction FFX was utilized in conjunction with concurrent gemcitabine and radiotherapy in patients with borderline resectable PDAC. Given that the trial's accrual goal was not met, the fact that this resection rate fell short of the threshold for statistical significance could have been a factor [9]. After neoadjuvant therapy, tumor resectability should be evaluated if pancreatic cancer was staged as unresectable at the time of the first diagnosis. Surgery should be performed on individuals with a high probability of R0 resection if they are in good enough health since it considerably improves their prognosis. Therefore, the notion of treatment for these patients should include surgery as a potential option. However, the technologies used today (CT and MRI) to determine the resectability of pancreatic cancer are not always accurate at predicting R0 resectability. In order to enhance R0 resectability assessments, new approaches must be sought after [10,11].

Adverse Effects

The course of treatment may be impacted by the unfavorable consequences of chemotherapy, as it is challenging and exhausting, and it can have a number of unpleasant side effects that make the patient unfit to continue the treatment. Pusceddu et al. reported the toxicities of FFX in comparison to GnP arms [12].

The two arms were compared for AEs of grades 3-4 (G 3-4) with hematological and non-hematological toxicities. In the GnP arms, neutropenia and febrile neutropenia were significantly reduced. As opposed to FFX, GnP treatment was more frequently associated with G 3-4 anemia. Only nausea and neurotoxicity showed a statistically significant difference between the two arms among non-hematological effects (90% less nausea and 2.5 times higher neurotoxicity with GnP as compared with FFX). Treatment time was comparable across GnP and FFX, lasting three (monthly) cycles as opposed to 5.4 (biweekly). There were no reports of toxic fatalities, dose reductions, or therapy halts [12]. The moderate benefit of these regimens should be weighed against the potential side effects, and for patients in poor general health, the best supportive care should be considered as a realistic alternative.

Immunotherapy for Pancreatic Cancer

By triggering, enhancing, or reducing the immune system's response, immunotherapy treats disease. Cancer immunotherapy aims to improve or restore the immune system's capacity to recognize and eliminate cancer cells by overcoming the processes through which malignancies thwart and suppress immune responses. Although immunotherapy has seen a therapeutic resurgence in recent years for many solid tumors, pancreatic cancer, one of the most immune-resistant tumor forms, did not show any substantial benefit with immunotherapy alone. Yet, there is still hope for effective treatments for pancreatic cancer, particularly for metastatic pancreatic cancer that is resistant to all current therapies. Vaccination, adoptive immunization, and immunological checkpoint blockade are currently the mainstays of pancreatic cancer immunotherapy [5].

Limitations of the study

One of the key limitations of our study is the relatively small number of studies included in our systematic review and the incomplete coverage of all aspects related to selecting chemotherapeutic regimens. Our study did not delve deeply into economic considerations, patient preferences, or emerging therapies. These unexplored aspects are important for a comprehensive understanding of treatment decisions. To address these limitations, future research should aim to include a larger and more diverse set of studies, encompassing a wider range of aspects related to selecting chemotherapeutic regimens. Additionally, studies that explore patient-centered outcomes and cost-effectiveness considerations would contribute to a more holistic understanding of this complex decision-making process.

## Conclusions

The outcomes of this study imply that using multiple medication regimens for advanced pancreatic cancer may optimize outcomes. Additionally, there are numerous chemotherapies that can be selected based on patients' clinicopathological backgrounds. In both the first-line and, to a lesser extent, the second-line settings, a number of multidrug regimens have encouraging potency and tolerable toxicity. When the corresponding regimens are given to patients in real-world settings, the outcomes reported in RCTs appear to be very consistent. Improving preventative tactics and early identification is crucial to enhancing surgical results in the fight against the formidable challenge of pancreatic cancer. Combination therapy, such as standard therapy (surgery, radiotherapy, or chemotherapy) combined with immunotherapy and target therapy, should also be effective in the treatment of pancreatic cancer. The success of combination therapy will be aided by improving our knowledge of the molecular basis of pancreatic cancer and discovering more potent, individualized systemic medicines. Therefore, more research in the fields of targeted and immunotherapy is required to achieve promising results in the future.
