# Correction: A Systematic Review of Chemotherapeutic Regimens Used in Pancreatic Cancer

**DOI:** 10.7759/cureus.c360

**Published:** 2025-10-14

**Authors:** Nimra Awais, Travis Satnarine, Areeg Ahmed, Ayesha Haq, Deepkumar Patel, Grethel N Hernandez, Kofi D Seffah, Mustafa Abrar Zaman, Safeera Khan

**Affiliations:** 1 Research, California Institute of Behavioral Neurosciences and Psychology, Fairfield, USA; 2 Pediatrics, California Institute of Behavioral Neurosciences and Psychology, Fairfield, USA; 3 Internal Medicine, California Institute of Behavioral Neurosciences and Psychology, Fairfield, USA; 4 Family Medicine, California Institute of Behavioral Neurosciences and Psychology, Fairfield, USA; 5 Internal Medicine, Piedmont Athens Regional, Athens, USA; 6 Internal Medicine, St. George's University School of Medicine, Newcastle Upon Tyne, GBR

This article has been corrected as follows:

- A footnote was added to Table 1 explaining that an additional 12 records were identified through supplementary searches.
- The PRISMA diagram was corrected to clarify that 225 records were identified after initial filtering.

